# Magnetophoresis in Centrifugal Microfluidics at Continuous Rotation for Nucleic Acid Extraction

**DOI:** 10.3390/mi13122112

**Published:** 2022-11-29

**Authors:** Sebastian Hin, Nils Paust, Markus Rombach, Jan Lüddecke, Mara Specht, Roland Zengerle, Konstantinos Mitsakakis

**Affiliations:** 1 Hahn-Schickard, Georges-Koehler-Allee 103, 79110 Freiburg, Germany; 2 IMTEK—Laboratory for MEMS Applications, Georges-Koehler-Allee 103, 79110 Freiburg, Germany

**Keywords:** centrifugal microfluidics, magnetophoresis, nucleic acid extraction

## Abstract

Centrifugal microfluidics enables fully automated molecular diagnostics at the point-of-need. However, the integration of solid-phase nucleic acid extraction remains a challenge. Under this scope, we developed the magnetophoresis under continuous rotation for magnetic bead-based nucleic acid extraction. Four stationary permanent magnets are arranged above a cartridge, creating a magnetic field that enables the beads to be transported between the chambers of the extraction module under continuous rotation. The centrifugal force is maintained to avoid uncontrolled spreading of liquids. We concluded that below a frequency of 5 Hz, magnetic beads move radially inwards. In support of magnetophoresis, bead inertia and passive geometrical design features allow to control the azimuthal bead movement between chambers. We then demonstrated ferrimagnetic bead transfer in liquids with broad range of surface tension and density values. Furthermore, we extracted nucleic acids from lysed *Anopheles gambiae* mosquitoes reaching comparable results of eluate purity (LabDisk: A260/A280 = 1.6 ± 0.04; Reference: 1.8 ± 0.17), and RT-PCR of extracted RNA (LabDisk: Ct = 17.9 ± 1.6; Reference: Ct = 19.3 ± 1.7). Conclusively, magnetophoresis at continuous rotation enables easy cartridge integration and nucleic acid extraction at the point-of-need with high yield and purity.

## 1. Introduction

In recent years, several centrifugal microfluidic systems for fully automated sample-to-answer nucleic acid analysis have integrated complex process chains including (i) lysis of pathogens or mammalian cells, (ii) nucleic acid extraction and purification and (iii) target nucleic acid amplification and detection [1,2,3,4,5]. Particularly for the process of extraction and purification, solid phase extraction based on the Boom chemistry has been widely used, namely the unspecific binding of free nucleic acids from a lysate onto silica surfaces of a solid phase, in the presence of high chaotropic salt concentrations [6,7]. After one or more washing steps in different liquids, nucleic acids can be eluted from the surface in an aqueous environment [8,9]. Despite the broad use of this extraction principle, it poses challenges especially concerning the selection and integration of the most appropriate solid phase for a particular application [10].

Silica particles with a magnetic core (magnetic beads) are frequently utilized as mobile solid phases. Nucleic acids are unspecifically bound to the beads’ surface and the mobile solid phase is moved from buffer to buffer solution by utilizing magnetic force. This process, termed magnetophoresis, can be achieved by positioning permanent magnets in the cartridge [11] or in the processing device [12,13], which provides several advantages. In terms of design, it does not require a valve or active microfluidic components for bead actuation. Furthermore, it exploits azimuthal space, thus overcoming the issue of radial space limitation induced by the directionality of the centrifugal force, and allowing the integration of workflows with high volumes while consuming comparatively low footprint. During cartridge assembly, a magnetic bead suspension can be easily dispensed by automated robotic stations to a chamber of interest in the cartridge and dried down. During the test, the beads can be resuspended on demand when they come in contact with liquid. Additionally, a broad collection of quality-controlled magnetic beads with diverse surfaces, core and geometric features is commercially available [14,15] that can be applied on extraction of nucleic acids for numerous applications, such as for detection of viruses, bacteria and parasites [16], single-nucleotide polymorphisms tissue homogenates [17], microRNA [18], and circulating tumor DNA (ctDNA) for early cancer detection [19]. Overall, these advantages can offer a drastic reduction of system complexity, while maintaining a high degree of integration that is required for sample-to-answer analysis.

The challenge in bead-based solid phase extraction is the handling of beads during their transfer from one chamber to another. In non-centrifugal microfluidic systems, recent work describes methodologies for focussing particles in microfluidic chambers and for generally predicting the motion of micro-scale entities under the influence of external forces [20]. Furthermore, Khashan et al., describes magnetic field-induced concentration and/or sorting of magnetic entities in a scalable way using a repulsion-based sorting method [21]. In centrifugal microfluidics, until now, the microfluidic disk had to be stopped in order for the beads to be attracted by a stationary magnet, pulled out of one liquid and flushed to the next chamber in a step-wise azimuthal transfer as accurate as 0.5° (gas-phase transfer magnetophoresis) [12,22]. Brassard et al., achieved a bead transfer during permanent rotation, but their configuration required external pressure pumps to timely route the liquid, thus, partly sacrificing compactness and full integration [23]. This procedure of stopping the rotation for magnetophoresis is suboptimal and dependent on the liquid’s wetting properties and contact angle with the cartridge. Furthermore, during the rotation interruption, and in the absence of centrifugal forces acting on the liquid, the capillary forces dominate and may easily lead highly wetting liquids such as the binding and washing buffers to creep along the chamber walls and edges. Lastly, the variability of the radial level of the liquid-gas interface renders the bead transport barely predictable and prone to bead loss. Consequently, this may lead to nucleic acid loss, suboptimal extraction and downstream sensitivity yields [11,12].

In this context, the goal of the present work was the development of a novel process for magnetophoresis under continuous rotation in centrifugal microfluidics, by precisely tailoring and controlling the interplay between centrifugal and magnetic forces. Centrifugal forces are easier to control than the capillary forces, while magnetic forces can be controlled by the design of a magnetic field. Thus, upon appropriate design, the interplay between magnetic and centrifugal forces within the rotating cartridge can create a well-controlled environment to enable robust and reproducible handling and transfer of beads during disk rotation. The present work includes (i) simulation, design and characterization of a magnetic field that exerts sufficiently high magnetic force on the magnetic beads to achieve magnetophoresis under rotation; (ii) investigation of microfluidic features such as structure design, liquid properties and centrifugal protocols that support the magnetophoresis and control the interplay between magnetic and centrifugal forces; and (iii) screening of beads with diverse magnetic properties, their interaction with the designed magnetic field and their impact on magnetophoresis.

The feasibility of the magnetophoresis under rotation was demonstrated through the extraction of nucleic acids from mosquito homogenates using quantitative parameters for quality control, such as the purity of eluates and the reproducibility of the nucleic acid extraction yield, which were compared with benchtop reference methods. This application scenario was selected because infections such as malaria, dengue and chikungunya that are derived from disease-carrying mosquitoes are responsible for more than 17% of all infectious diseases, causing more than 700,000 deaths annually [24], while the co-management of these common diseases between humans and mosquitoes is crucial for the global One Health initiatives [25]. The development of insecticide resistances [26,27] of such mosquitoes is an emerging threat, which needs to be studied, detected and monitored at molecular level in order to tailor the actions of insecticide and infection control programs [28,29].

## 2. Magnetophoresis at Continuous Rotation

**Figure 1:**
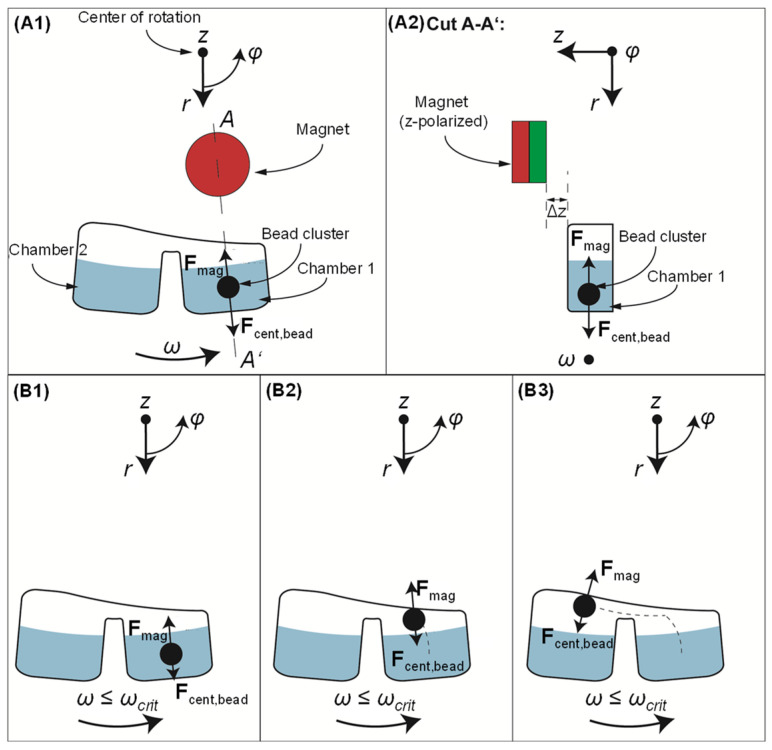
Schematic of the principle of gas-phase magnetophoresis under rotation. (**A1**): Two microfluidic chambers are located radially outwards from a magnet. During rotation, the magnetic beads undergo the centrifugal force, ${\vec{F}}_{\mathrm{cent},\mathrm{bead}}$ and the magnetic force, ${\vec{F}}_{\mathrm{mag}}$ (notably, only the radial component of the ${\vec{F}}_{\mathrm{mag}}$ vector is indicated here, as it is the one that influences the magnetophoresis between the chambers). The A-A’ cut (**A2**) shows the distance, Δ*z,* between the magnet and the top of the cartridge, in the *z*-direction. (**B1**,**B2**): When the rotation takes place at an angular velocity below a critical value, *ω*_crit_, the magnetic force is higher than the centrifugal force, thereby the pellet is pulled radially inwards. (**B3**): Azimuthal transfer of the magnetic beads to a downstream chamber. This movement is supported by the bead cluster inertia depending on the direction of rotation.

The operating principle is described in Figure 1. The basic set up involves (i) a rotating cartridge, indicatively consisting of two azimuthally arranged and connected chambers with liquid at the same radial level; (ii) magnetic beads immersed in the chamber forming a pellet under the influence of the magnetic field; and (iii) a permanent magnet at a fixed position, radially inwards from the azimuthally arranged chambers where the magnetic beads are moved, and at a vertical distance Δ*z* above the rotating cartridge.

Due to the geometric configuration of the set up, we use cylindrical coordinates (*r*, *φ*, *z*) to describe the force vectors and components. Upon rotation, the forces exerted on the magnetic beads are: (i) the centrifugal force, ${\vec{F}}_{\mathrm{cent},\mathrm{bead}}$, whose vector points away from the center of rotation and has only a radial component with magnitude:

(1) $$F_{\mathrm{cent},\mathrm{bead}}=\Delta m\;\omega^{2}\;r=(\rho_{\mathrm{bead}}-\rho_{\mathrm{liqu}})\;V_{\mathrm{bead}}\omega^{2}r$$

where, *ω* is the cartridge’s angular velocity, *r* is the radial distance from the center of rotation, *V*_bead_ is the bead cluster volume, and *ρ*_bead_ and *ρ*_liqu_ are the densities of the bead pellet and the surrounding fluid, respectively; (ii) the magnetic force, ${\vec{F}}_{\mathrm{mag}}$, whose vector points towards the magnet and has all three coordinate components:

(2) $${\vec{F}}_{\mathrm{mag}}=V_{\mathrm{bead}}\;\frac{\chi_{\mathrm{mag}}-\chi_{\mathrm{liqu}}}{{\text{µ}}_{0}}\;(\vec{B}\cdot \nabla )\vec{B}$$

where *χ*_mag_, *χ*_liqu_ are the magnetic susceptibilities of the magnetic material in the bead and its surrounding medium, respectively (the latter being negligible as it is at least five orders of magnitude lower than the *χ*_mag_, for the liquids that we consider as surrounding media [30,31,32]), and μ_0_ is the vacuum permeability [33,34]. $\vec{B}$ is the magnetic flux density vector and $\left(\vec{B}\cdot \nabla \right)$ is the gradient of the magnetic flux density. Equation (2) is applicable as long as the magnetic beads’ magnetization is at saturation [33]; and (iii) the surface force,$\;{\vec{F}}_{\mathrm{surf}}$ that applies when the bead pellet is about to exit the liquid (not shown in Figure 1), and whose vector points towards the center of rotation with only a radial component and magnitude:

(3) $$F_{\mathrm{surf}}=6^{\frac{1}{3}}\;\;\pi^{\frac{2}{3}}\;\sigma_{\mathrm{liqu}}\;V_{\mathrm{bead}}$$

based on a model utilized in previous work [12,35], where *σ*_liqu_ is the surface tension of the liquid. During rotation, there is an interplay between the aforementioned forces (friction/resistance forces upon the bead pellet during its motion in the liquid and before reaching the liquid/air interface are considered negligible). The net integral magnetic force over one entire rotation, *F*_mag,net_, will define the direction of the bead cluster motion at a specific radial distance *r* (orbit) and angular velocity *ω* (Equation (4)).

(4) $$F_{\mathrm{mag},\mathrm{net}}\left(\omega ,r,z\right)=\frac{\int_{0{}^{\circ}}^{360{}^{\circ}}F_{\mathrm{mag}}\;d\varphi }{360{}^{\circ}}$$

If the net magnetic force per round is higher than the centrifugal force, i.e., when the angular velocity is below a critical value, *ω*_crit_, the magnetic bead pellet will move radially inwards.

## 3. Materials and Methods

### 3.1. Permanent Magnet Setup

**Figure 2:**
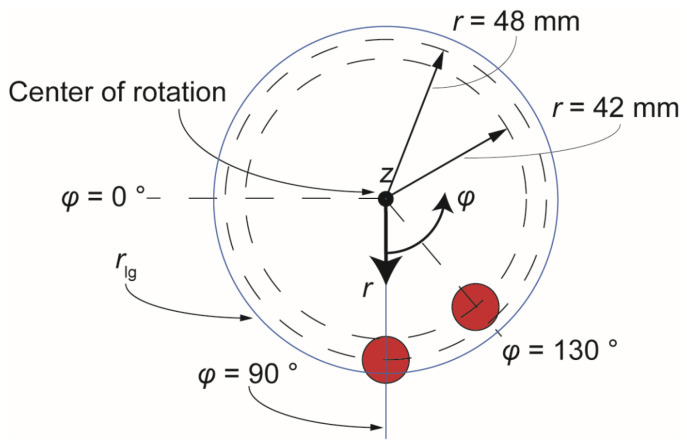
Basic arrangement of magnets under investigation.

The magnets used in this work were cylindrical (diameter 15 mm, height 5 mm) NdFeB permanent magnets (Webcraft GmbH, Gottmadingen, Germany), axial magnetization, magnetization strength ‘SH45’, coating with Ni-Cu-Ni and maximal temperature tolerance 150 °C. Using a previous setup by Strohmeier et al. as a starting point [12], the magnets were positioned above the cartridge so that their bottom circular surface was 1.5 mm above the surface of the highest microfluidic chamber (4 mm high). Thus, the maximum *z*-distance between the bottom of the magnet and the bead cluster was 5.5 mm and 1.5 mm when the beads were located at the bottom or at the top of the chamber, respectively. The *r*-*φ* plane of *z* = 0 is considered to be the plane of the disk cartridge. Furthermore, the radial distance of the center of the magnets from the center of rotation was *r*_1_ = 42 mm and *r*_2_ = 48 mm. The azimuthal spacing between the magnets was *φ* = 40° (Figure 2).

### 3.2. Simulation of the Magnetic Force

The simulation of the magnetic field, force and flux density was done using the Finite Element Model (FEM) package from COMSOL^®^ Multiphysics. The quantitative parameters used for the simulation were: *V*_bead_ = 4 mm^3^, *χ*_mag_ = 0.29 (a value used before in the case of magnetic beads) [12], *ρ*_liqu_ = 883 kg m^−3^, and *ρ*_bead_ = 1643.5 kg m^−3^, the latter being approximated assuming a composition ratio of 20% maghemite (*ρ* = 4860 kg m^−3^) as magnetic core and 80% SiO_2_ as outer shell (*ρ* = 265 kg m^−3^) [36]. The permanent magnet remanent magnetic flux density was 1.32 T according to the manufacturer [37]. Computational analysis for the initial, basic arrangement of magnets as shown in Figure 2, provides in Supplementary Figure S1 the magnetic vector potential *ψ* (in A), and the absolute magnetic flux density *B* at the *r*-*φ*-plane of the coordinate system at *z* = 3 mm, i.e., at a distance of 2.5 mm from the magnets. This helps to comprehend the motion of a magnetic particle under the influence of the field.

### 3.3. Measurement of Magnetic Flux Density

The magnetic flux density was measured by means of a three-axis teslameter (3MTS, Senis AG, Baar, Switzerland), which was not movable, while the magnets were arranged to rotate. The teslameter was mounted on the chuck of a micro-milling machine (Evo CNC machine, KERN Microtechnik GmbH, Eschenlohe, Germany) and was measuring the flux density. The magnets were fixed on a PMMA disk, which was attached on the spindle of the CNC machine and could rotate at 2 Hz. Only one pair of magnets was used, for simplicity (configuration of Figure 2). In *z*-direction (range between 2 mm and 3.5 mm) and in *r*-direction (range between 45 mm and 60 mm), the flux density was measured in 0.5 mm steps, while in *φ*-direction (range between 0° and 360°) in 0.18° steps.

### 3.4. Measurement of Magnetic Bead Cluster Velocity

Rectangular microfluidic chambers, 3 mm deep, were designed on a disk, with line markers next to them (at 2 mm step) to enable the observation of the bead cluster position over time. The setup is shown in Supplementary Figure S2. Teflon coating was applied along each chamber in order to render it representative of the conditions that the magnetic beads face in the actual extraction structure. The chambers were filled with liquid and magnetic bead suspension. The disk was rotated below the magnets and the position of the beads was recorded at fixed intervals by using a stroboscopic camera (capturing one image per turn). The images depicting the position of the bead cluster were analyzed using the Fiji plugin [38]. The speed of the bead cluster, *V*_bead_ that travels along a microfluidic chamber filled with the relevant buffer is the main parameter to be extracted with this experiment and was calculated via the change of the bead cluster position between two images, as *V*_bead_ = Δ*r*(*t*)/Δ*t*. The buffer properties were also measured for the determination of the bead cluster velocity. The liquid density, *ρ*_liqu_, was measured by weighing specific volumes of liquids (500 μL) using a high precision scale (Adventurer, Ohaus Europe GmbH, Greifensee, Switzerland). The surface tension, *σ*_liqu_, was measured using pendant drop tensiometry on a contact angle measurement device (OCA15EC, DataPhysics Instruments GmbH, Filderstadt, Germany) [35]. Buffer kits based on the Boom chemistry [6] were used from three representative suppliers (Supplementary File S3), namely: (i) the MagSi DNA mf kit from MagnaMedics Diagnostics BV (currently magtivio BV, Nuth, the Netherlands), (ii) the Innuprep MP basic A from Analytik Jena GmbH (Jena, Germany), and (iii) the Silane viral NA kit from Thermo Fisher Scientific (Waltham, MA, USA). Apart from single buffers, measurements were also conducted on mixtures of, for example, lysis and binding buffers, given that magnetic beads must be pulled out of such liquid mixtures. Since in a bead-based extraction, several different buffers are involved with diverse liquid properties, for this experiment we used a representative liquid consisting of 50% (*v*/*v*) ethanol in water, with density ~883 kg m^−3^ and a surface tension of ~31.25 mN m (a summary of all used buffers is provided in Supplementary File S4).

### 3.5. Manual Nucleic Acid Extraction

The manual nucleic acid extraction from the mechanically lysed mosquito pools (Supplementary File S5) was performed as a benchtop reference to the LabDisk extraction, using the ‘MagSi-DNA mf’ kit (MagnaMedics Diagnostics BV—currently magtivio BV, Nuth, the Netherlands) [39]. The protocol was as follows:

- Mixing of 150 μL lysis buffer and 200 μL sample;
- Incubation for 10 min at 350 rpm and 35 °C on a thermo mixer;
- Addition of 440 μL binding buffer and 20 μL magnetic beads;
- Collection of the beads for 3 min on the magnetic separator rack (‘MM12+12’, MagnaMedics Diagnostics BV—currently magtivio BV, Nuth, the Netherlands) in order to remove the supernatant;
- For the first washing step, addition of 200 μL washing buffer 1 into the tube;
- Incubation of the mixture for 2 min at 350 rpm and 35 °C on a thermo mixer;
- Separation of the beads for 3 min so that the supernatant could be pipetted out;
- For the second washing step: repetition of the same steps and volumes as washing step 1 was carried out (except that washing buffer 2 was used);
- For the elution step, addition of 180 μL elution buffer into the tube;
- Incubation of the mixture for 10 min at 350 rpm and 50 °C on a thermo mixer;
- Separation of the beads for 3 min on the magnetic separator rack;
- Pipetting out of the eluate and storage at −20 °C.

### 3.6. Manual Nucleic Acid Amplification on the Eluates

Manual real-time reverse transcription polymerase chain reaction (RT-PCR) of the mosquito RPS7 control gene was carried out using a RotorgeneQ (QIAGEN GmbH, Hilden, Germany) as follows:

- Adjustment of the primer/probe concentrations at 100 nM RPS7_F, 200 nM RPS7_R and 250 nM RPS7_P;
- Mixing of the RPS7 primers with DNase/RNase-free water to a total volume of 9 μL;
- Addition of 1 μL eluate into this mixture, using a 1/16 fraction of a lyophilized amplification pellet (lyocake) per reaction (customized pellet including reverse transcriptase, Taq-Polymerase, Mg^2+^, nucleotides, buffer salts; GE Healthcare UK Limited, Chalfont St Giles, UK/Fast Track Diagnostics, Luxemburg);
- Realization of thermocycling under the following conditions: 5 min RT-step at 50 °C, 60 s initial denaturation at 95 °C, thermocycling 40 × (95 °C, 10 s and 60 °C, 60 s);
- Signal readout was done using the green channel;
- Data analysis was done with the Rotorgene Software (QIAGEN, Hilden, Germany), using the ‘dynamic tube’ and ‘slope correct’ filters. The first ten PCR cycles were not taken into account during baseline calculation. The threshold was manually set to 0.00686.

### 3.7. Spectrophotometric Analysis of the Eluates

Spectrophotometric analysis (spectrophotometer NanoDrop One^TM^, Thermo Fisher Scientific Inc., Waltham, MA, USA) was carried out on the eluates derived from the LabDisk and the manual extraction in order to deduce their purity. From all eluates, an aliquot of 2 µL was used to measure the absorbance at 260 nm (A260) and 280 nm (A280), representing the nucleic acid and protein concentration in the eluate, respectively. Thus, the ratio A260/A280 gives the relative contamination of the eluate with proteins. An elution buffer (10 mM Tris-HCl, pH 8.8) was used as a blank reference.

### 3.8. LabDisk Cartridge

For performing the automated nucleic acid extraction, the disks were processed on a LabDisk Player 1 functional model instrument (QIAGEN Lake Constance—currently Dialunox GmbH, Stockach, Germany). The disk was mounted on the device, the sample was pipetted in the inlet, the inlet was sealed and the developer mode software processed the microfluidic and temperature protocol, as summarized in Supplementary File S6. Details on the fabrication of the LabDisk cartridges are provided in Supplementary File S7.

## 4. Results and Discussion

### 4.1. Definition of the Critical Frequency for Magnetophoresis under Rotation

In order to determine the critical frequency at which bead transport under rotation starts to occur, we first examine the forces that are exerted on a bead pellet at three different distances from the center of rotation, as indicative positions of the liquid-gas interface, *r*_lg_ (45 mm, 52 mm, 59 mm). This is a typical space of motion of the magnetic beads through magnetophoresis within microfluidic chambers for nucleic acid extraction. At these three representative liquid levels, a qualitative representation of the directionality of the magnetic force vectors is shown in Supplementary Figure S3a and a simulated calculation of the magnetic force magnitude in the radial direction, *F*_mag,r_, for every value of *φ* from 0° to 180° is shown in Supplementary Figure S3b. For *r* = 45 mm and 52 mm, force peaks appear at positions *φ* = 90°, 100°, 120° and 140° and values higher than 7 mN in the radial direction can be generated along an azimuthal range of about 70°–170°. For *r* = 59 mm, these peaks are clearly lower, with *F*_mag,r_ < 1 mN.

The net magnetic force (integral over one entire rotation, Equation (4)) radial- and *z*-components are calculated for specific radial positions (orbits) as shown in Supplementary Figure S4. Interestingly, the results show low values of the radial component of the force (<1 mN). Given the fact that a bead cluster of 4 µL volume needs to overcome a surface force of ~0.1 mN in order to leave from a solution of 50% (*v*/*v*) ethanol, and ~0.5 mN in case of pure water, the above force is marginally not sufficient for magnetophoretic bead transfer; this is also according to values reported in prior work (4.5 mN) [12]. However, the *z*-component of the net magnetic force can reach up to 3 mN for an orbit at 45 mm.

**Figure 3:**
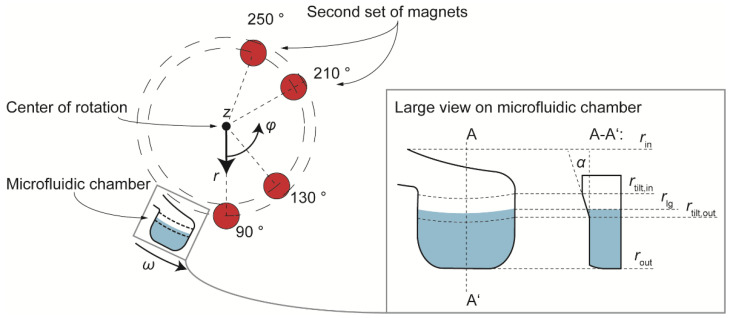
(**Left**): The updated configuration (and the final one, compared to Figure 2) in which a second pair of magnets is added in order to enhance the area over a full rotation along which magnetic force is exerted on the magnetic beads. (**Right**): A special shape of the microfluidic chamber in which an inclined top side of the chamber between *r*_tilt,in_ and *r*_tilt,out_ enables the *z*-component of magnetic force to contribute to the radial bead motion.

Due to this high value of *F*_mag,z_ a special design of the chamber was adopted in which its wall facing radially inwards was tilted by an angle α = 20° versus the horizontal plane, at the position of the liquid-gas interface. This shape enables the *z*-component of the magnetic force to contribute to the radial movement of the magnetic beads: $F_{\mathrm{mag},z\to r}=F_{\mathrm{mag},z}\times \tan^{-1}\left(\alpha \right)$, where $F_{\mathrm{mag},z\to r}$ represents the contribution of the magnetic force in *z*-direction to the radial magnetic force. This holds true for the radial range of the tilted chamber lid at *r* = [*r*_tilt,in_, *r*_tilt,out_], as shown in Figure 3, right. The figure (left) also shows an updated configuration with two additional magnets, which aims to enhance the magnetic influence on the bead cluster over a longer azimuthal range by installing the second pair of magnets at an azimuthal distance of 80° from the first pair. The installation of the magnets on the lid of the LabDisk processing device is shown in Supplementary Figure S5.

**Figure 4:**
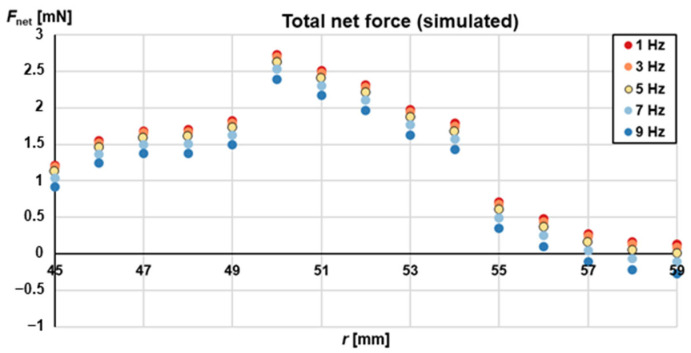
Net (simulated over one full rotation) total (magnetic, centrifugal and surface) force in *r*-direction plotted for a range of different radial positions and for various rotational frequencies. The plot shows the simulated force according to Equation (5). The net force increases in general, if the rotational frequency is decreased. In addition, the geometry shown in Figure 2 leads to an additional net force in the range of the tilted chamber wall, caused by the term *F*_mag,z→r_. Below the magnet at low radial positions, the net force decreases due to lower magnetic gradient in this area.

Under these new conditions, the total, net radial component of the magnetic force can reach up to 2.7 mN (Supplementary Figure S6). This is considered sufficient to contribute to the gas-phase magnetophoresis [12]. In principle, the force could be further increased if the distance between the lower circular surface of the magnet and the highest surface of the disk would be reduced (currently at 1.5 mm; previous work reports 0.5 mm distance). However, due to high rotational frequencies of >40 Hz that are required during the bead-based extraction protocol for some unit operations such as pneumatics and buffer release from stickpacks [40], we preferred to keep the 1.5 mm distance for safety reasons, which leads to the forces calculated above. For the determination of the critical frequency at which the magnetophoresis takes place, we take into consideration all forces that are exerted on the magnetic beads (Equations (1)–(3)) and integrate over one full rotation (*φ* = 0°–360°), resulting in Equation (5).

(5) $$F_{\mathrm{net}}\left(\omega ,r,z\right)=\frac{1}{360}\;\int_{0}^{360}F_{\mathrm{mag},r}\;d\varphi +y_{1}\int_{0}^{360}\;F_{\mathrm{mag},z\to r}d\varphi -F_{\mathrm{cent},\;\mathrm{bead}}-y_{2}\;F_{\mathrm{surf}}$$

where *y*_1_ = 0 outside the [*r*_tilt,in_–*r*_tilt,out_] (in our case [50 mm–54 mm]) and *y*_2_ = 0 outside the *r* = *r*_gl_ (in our case 52 mm). This total net force, *F*_net_, simulated, is shown in Figure 4 plotted against radial position and for various rotating frequencies. As it appears, for frequencies *f* ≤ 5 Hz the total net force is positive (i.e., pointing radially inwards) along the full range of radial distances. This is a decisive factor as it will ensure an unobstructed magnetophoresis.

Beyond the simulation-based results, the magnetic flux density was also measured experimentally, in order to calculate the magnetic force and consequently, the total net force. Deviations were observed compared to the simulation: the experimentally measured values of the magnetic force in radial direction *F*_mag,r_ for every azimuthal position *φ* were lower than the simulated ones (Supplementary Figure S7). There could be several possible explanations for this: (i) The gradient of the magnetic flux is strongly increased on the magnet’s edges, and depends on the edge geometry. In a simulation it is assumed that the magnet has an ideal geometry with sharp edges, while in reality it has a fillet-shaped radius of 470 µm (measured, *n* = 5), which can partly account for the observed experimental deviation. (ii) The fact that the experimental measurements were done when the magnets were rotating (while assumed static during simulation) may have led to peak flux densities not being measurable by the teslameter, thereby missing single peak values. (iii) During magnet mounting on the experimental setup, minor positioning deviations may have led to deviation of the experimental measurements versus the simulation. (iv) There may be manufacturing-dependent magnetization inhomogeneities along the *z*-direction.

The net force (integral over one entire rotation) calculated from the experimentally measured magnetic flux based on Equation (5) is shown in Supplementary Figure S8. The comparison between simulation and measurement data shows deviations of the magnetic force for radial positions between 55 and 57 mm, and for 50 mm, 47 mm and 45 mm. As possible reasoning, apart from the rationale explained before regarding the possible deviations between the simulated and experimental magnetic flux, we can assume an imperfect orientation of the magnets along the *z*-direction (slight tilting of the magnets in the *r*-*φ* plane), which could result in a tilting of the magnetic flux density streamlines. Thus, high magnetic force may appear at positions where it is not expected, since an increased gradient would be obtained if tilted field lines get in close proximity to each other. Despite the deviations though, the critical frequency was still determined to be at 5 Hz through the experimental calculations, similarly to the simulation, as it appears from Supplemenary Figure S8, where for rotational frequency *f* ≤ 5 Hz the net force is positive for the entire radial range of 45 to 59 mm.

### 4.2. Impact of Magnetic Bead Properties on Magnetophoresis under Rotation

**Figure 5:**
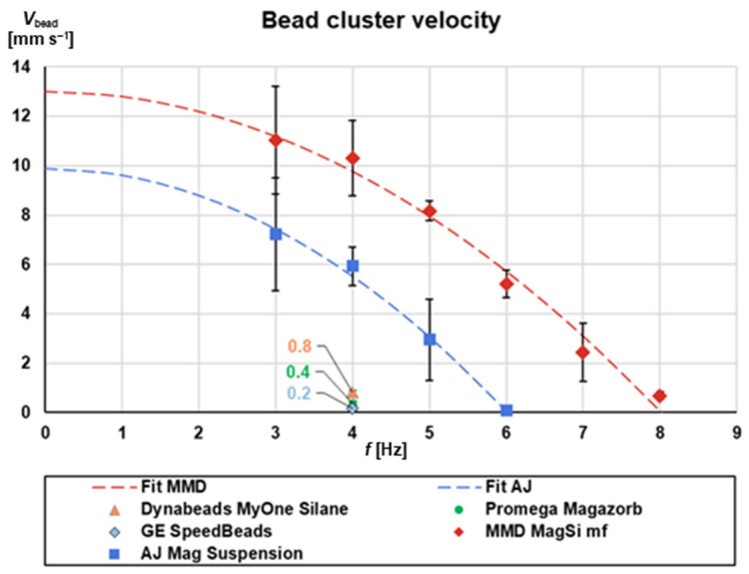
Bead cluster speed versus rotational frequency for different commercially available magnetic beads. AJ: Analytik Jena; MMD: MagnaMedics Diagnostics BV (currently magtivio BV); GE: GE Healthcare. The fit MMD and fit AJ gave R^2^ = 0.9837 and R^2^ = 0.9928, respectively (*n* = 3; 1× standard deviation). Ferrimagnetic beads respond very strong to the magnetic field setup. We attribute this to the clustering behavior of ferrimagnetic beads under an external magnetic field.

This section focuses on the material properties of the magnetic beads, their influence on the magnetic force (Equation (2)) and consequently on the magnetophoresis under rotation. The key parameters are the magnetic cluster volume, *V*_bead_, and the magnetic susceptibility *χ*_mag_. However, they are both difficult to define analytically [41] for the system of clustering magnetic beads that we examine because: (i) not their entire volume is magnetic due to their silica shell; (ii) the percent of magnetic material may vary from bead to bead and is often not disclosed by the manufacturers; (iii) the bead size and volume follows a distribution pattern based on manufacturing conditions, while the cluster size may also vary due to magnetic interactions between beads themselves. Therefore, instead of analytically calculating *V*_bead_ and *χ*_mag_, for the magnetic force calculation, we used a simple experimental setup for characterizing the suitability of selected magnetic beads, in combination with specific buffers, for magnetophoresis under rotation (Section 3.4 and Supplementary Figure S2). The correlation between the velocity of the magnetic bead cluster, *V*_bead_, and rotational frequency was experimentally assessed for five sets of beads (Figure 5). This workflow can be generic and serve as a blueprint for future screening of bead-based extraction kits for this purpose.

Interestingly, we observe two categories of response. Three bead types, namely Magazorb (Promega, Walldorf, Germany), Dynabeads MyOne Silane (Thermo Fisher Scientific, Waltham, MA, USA) and SpeedBeads (GE Healthcare, Chalfont St Giles, UK), exhibit almost no response to the presence of the magnetic field. Practically, they barely move under the influence of the magnetic field and for any of the examined rotational frequencies, as their *V*_bead_ values indicate. This may be attributed to their paramagnetic properties, i.e., they do not magnetize to saturation in the presence of a magnetic field, while they also tend to not create clusters. Therefore, it was judged that these beads are not appropriate for continuous rotation magnetophoresis.

On the other hand, the two bead sets, MMD MagSi mf and AJ Mag Suspension, exhibited a dependence of the *V*_bead_ from the rotational frequency that was simulated using a polynomial equation *V*_bead_ ~ *a* + *b* * *f*^2^, where *a* and *b* represent the cluster velocity contributions of the magnetic and centrifugal forces, respectively. The frequency *f*_0_ at which the beads start to move radially inwards can be deduced when setting *V*_bead_ = 0 and was calculated at 6 Hz for AJ Mag Suspension and at 8 Hz for MagSi mf beads. Since these values are higher than the critical frequency calculated in the previous sections, both these bead sets are candidates for being used in magnetophoresis under rotation. Notably, they both exhibit ferrimagnetic, instead of (super-) paramagnetic, properties. Ferrimagnetic beads strongly cluster under an external magnetic field. Since the magnetic force on a particle depends on the volume of magnetic material in the particle, the force on a beads cluster is larger than on a single bead. This is how we explain the strong response of ferrimagnetic beads compared to paramagnetic. In our subsequent experiments we selected the MagSi mf beads due to the faster response of *V*_bead_ to the rotational frequency.

### 4.3. Microfluidic Design and Protocol Supporting Magnetophoresis under Rotation

**Figure 6:**
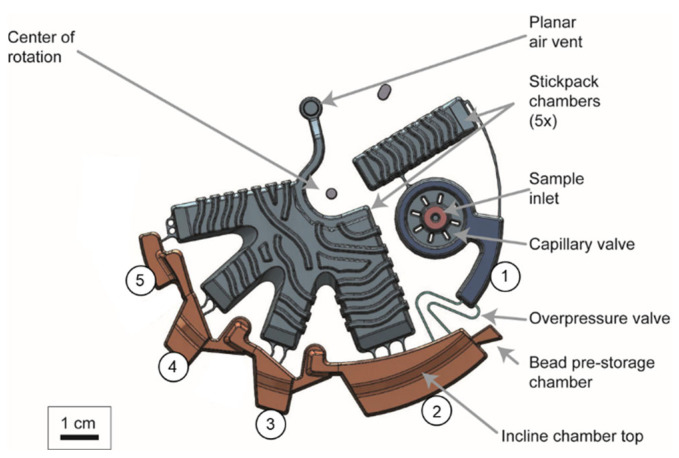
Design of the nucleic acid extraction microfluidic module. #1: Lysis chamber. #2: Binding chamber. #3: Washing chamber 1. #4: Washing chamber 2. #5: Elution chamber. Stiffening structures that provide higher mechanical stability are visible on top of the large structures that host the stickpacks. The chambers #2–5 were designed according to the findings from simulation, i.e., with tilted top chamber walls and radial positions to achieve magnetophoresis at the determined critical frequencies.

Following the simulations and calculations in the previous sections regarding critical parameters for continuous rotation magnetophoresis, the latter is further supported by specific microfluidic features of the bead-based extraction module (Figure 6). The sample is inserted in a dedicated inlet, which provides a flat ring structure, 0.5 mm deep, holding back wetting samples by capillary force, according to a previously demonstrated principle [42,43,44]. The sample is then transferred to the lysis chamber #1 by centrifugation but does not yet flow into the binding chamber #2, as the bypass of the overpressure valve connecting the lysis and the binding chamber is open, thus, preventing valve actuation. The next step is buffer release, during which the stickpacks [40] containing all buffers burst-open at 70 Hz, supported by heating at 60 °C in order to weaken the burstable sealing and release the liquids into the corresponding radially outwards chambers #1 to #5. Thus, lysis takes place using unidirectional shake-mode [45] for mixing the lysis buffer and the sample. The lysate is then transferred to the chamber #2 to mix with the binding buffer and the rehydrated magnetic beads. Subsequently, the magnetic bead cluster is transported through magnetophoresis under rotation into the washing chambers #1 and #2, and finally into the elution chamber. There, upon mixing in aqueous conditions at 60 °C for 10 min, nucleic acids are released from the beads into the eluate. The beads are then transported back to chamber #4 and the eluate is collected from chamber #5 and stored at −20 °C for further analysis.

**Figure 7:**
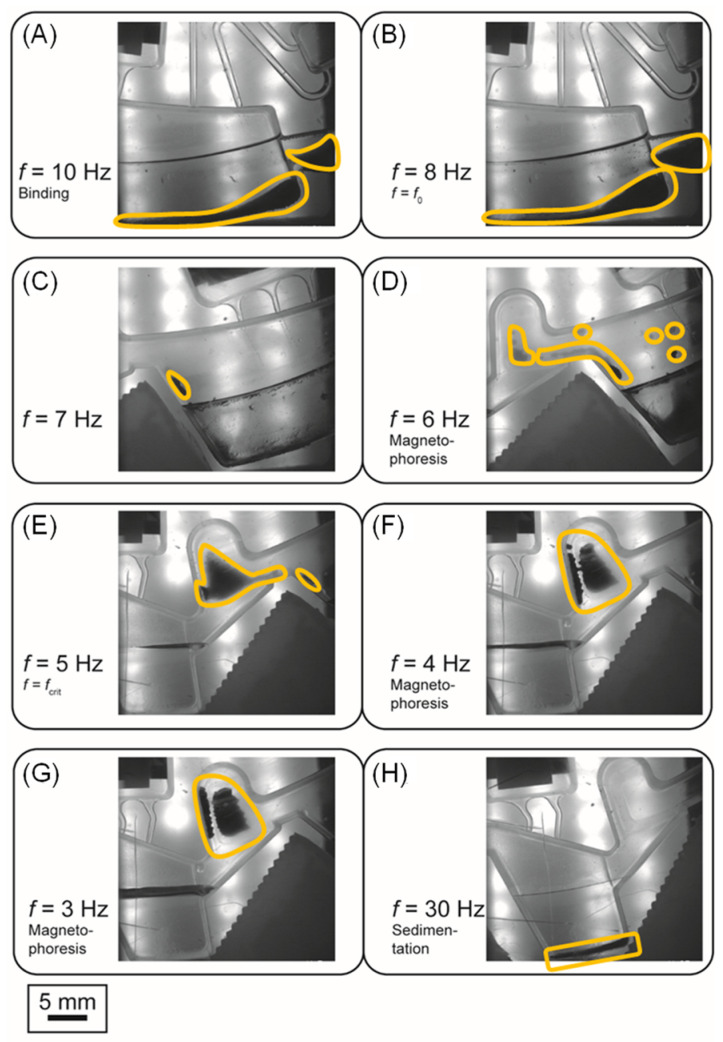
Stroboscopic images indicating the steps of continuous rotation magnetophoresis. The yellow frame marks the bead cluster. (**A**): Magnetic beads are rehydrated and remain in the binding chamber. (**B**)–(**G**): Stepwise reduction of the rotational frequency in order to collect the beads, create a cluster, pass the liquid-air interface and achieve magnetophoresis. In (**G**) the cluster is blocked by the pocket above washing chamber 1 in order to prevent further proceeding azimuthally. (**H**): Upon rotation at 30 Hz the cluster is sedimented in the subsequent chamber (this case, washing chamber 1).

The microfluidic design and protocol features that support the magnetophoresis are discussed in more detail below. In the binding chamber, alternating rotational frequencies between *f* < *f*_0_ and *f* >> *f*_0_ are applied in order to facilitate efficient mixing of the magnetic beads in the lysate/binding buffer, by permanent inward-outward movement through convective mixing [46]. The mixing also includes *z*-direction motion of the beads under the influence of the *z*-component of the magnetic force. After the binding process ends, the frequency is reduced to *f* ≤ *f*_0_ (*f*_0_ = 8 Hz, as calculated in Section 4.2) so that the beads start to move through the liquid towards the liquid-air interface. A stepwise reduction of the rotation frequency from *f*_0_ to *f*_crit_ = 5 Hz for magnetophoresis is applied, leading to stepwise magnetophoresis of the bead cluster. This is advantageous over instantaneous bead transfer because we avoid the generation of a liquid plug that could cause liquid crosstalk and contamination to the next chamber. Thus, the applied protocol was the reduction of the frequency from *f*_0_ to *f*_crit_−2 Hz in steps of 1 Hz, thereby leading to a controlled transfer through the liquid-gas interface (Figure 7). The *f*_crit_−2 Hz is selected to obtain a process, where not all beads cluster at a time. In case many beads are suspended, the instant setting of *f*_crit_ would lead to sudden clustering, which can cause splashing if a large cluster leaving the liquid is generated.

Once the beads are at the air phase, their azimuthal transfer to the next chamber is done based on the inertia of the magnetic bead cluster by controlling the direction of rotation (Supplementary Figure S9). At the end of the azimuthal magnetophoresis ‘neck’ between two chambers, a bead pocket is structured that collects the beads and prevents them from further azimuthal move. Then, the bead cluster is ‘flushed’ towards the corresponding chamber that is radially outwards from the bead pocket using a rotational frequency much higher than the *f*_crit_, at this case 30 Hz, in order to ensure it fully passes through the air-liquid interface. Notably, the design of the microfluidic structure prevents part of the bead cluster from ‘returning’ to its preceding chamber. Manufacturing-wise, a special milling procedure of the thermoforming tool allowed the reduction of distance between milling lines, thereby the reduction of groves where small bead clusters could potentially be trapped and lost (Supplementary Figure S10). The above steps are then repeated until the beads are collected in the elution chamber.

### 4.4. Nucleic Acid Extraction Using Magnetophoresis under Rotation

The application scenario for testing the magnetophoresis under rotation was the extraction of nucleic acid from mosquito homogenates, in the broader context of identification of insecticide resistance mechanisms through the expression levels of metabolic detoxification genes (RNA-level) [47,48]. This is typically performed through the quantitation of the expression of certain genes compared to a reference gene. In our work we used the reference gene RPS7 for demonstration of our developed set up, as this gene is a constantly expressed normalizer between different individuals [49,50,51].

**Figure 8:**
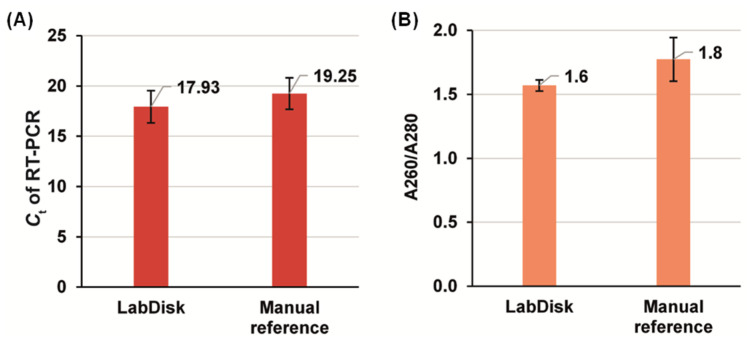
Results comparing the performance of the nucleic acid extraction using the LabDisk (*n* = 4) and the manual reference method (*n* = 3). (**A**): Ct values of RT-PCR on RPS7 gene (PCR on all eluates, derived from both the LabDisk and manual extraction, were performed in tube). (**B**): Spectrophotometric determination of eluate purity by means of protein contamination assessment.

Ten-mosquito pools were manually homogenized (ground) ex situ, as it has been shown by Mavridis et al. [17] that the use of mosquito-pools saves time, reduces costs, and allows for more efficient resistance allele screening than in the case of individual mosquito testing. One aliquot was tested with the disk and a second with the manual reference extraction method. At the end of each procedure, the eluates were analyzed in terms of protein contamination and the Ct values derived from real-time RT-PCR. These parameters were used in order to characterize the performance of the microfluidic-based extraction using bead magnetophoresis under rotation. The results are summarized in Figure 8.

The RT-PCR on the eluate derived from the LabDisk and manual reference gave an average Ct of 17.93 ± 1.62 (*n* = 4) and 19.25 ± 1.56 (*n* = 3), respectively. The absolute value of the LabDisk/Reference difference was 6.8%. In comparison with earlier work by Strohmeier et al., who had used magnetophoresis without continuous rotation on a centrifugal microfluidic cartridge and the deviation between LabDisk and reference was of the order of 30% (referring to recovered number of DNA copies [12]), our approach is a significant improvement. Notably, the present experiments need further extension in order to draw a final conclusion on the performance compared to prior work because a final assessment of a method for nucleic acid extraction and amplification is only possible when using exactly the same samples and nucleic acid targets. Importantly, the lower Ct (better performance) from the LabDisk than the reference may be attributed to the automation of processing in contrast to the manual method. The coefficient of variation (CV) between the two methods was also comparable (9.0% and 8.1% with the LabDisk and the manual method, respectively), which demonstrates the reproducibility of magnetic bead handling throughout the automated LabDisk process. In terms of protein contamination, which is assessed through the absorption ratio at 260 nm and 280 nm (A260/A280) to provide the relative contamination of the eluate with proteins, the values of 1.6 ± 0.04 and 1.8 ± 0.17 for the disk-based and reference-based extraction, respectively, shows a small deviation. Ideal values are of the order of 1.8–2.0 [52], which, however is not too far from the LabDisk-derived value. It indicates that the newly developed magnetophoresis approach performs in a satisfactory level, and that there is room for a further development in relation to the binding buffer and beads surface composition for better selectivity of nucleic acids binding over proteins. Further development could also focus on washing buffers composition for more efficient purification.

## 5. Conclusions

In this work we developed and demonstrated a process for magnetophoresis of magnetic beads under continuous rotation of a disk-shaped microfluidic cartridge. We investigated all parameters that are involved in this process, namely the magnetic field, the bead and buffer properties, as well as the centrifugal design and protocol. We defined a critical frequency, *f*_crit_ = 5 Hz, at which magnetophoresis under rotation occurs. It was concluded that ferrimagnetic beads are suitable for our scope, while (super-)paramagnetic are not. In this regard, a simple and broadly applicable experimental setup was developed to assess the suitability of a set of magnetic beads and buffers for continuous magnetophoresis under rotation, based on the bead cluster velocity versus the rotational frequency. Special microfluidic design features played a crucial role in magnetophoresis, namely (i) the tilted chamber top walls that enable the radial component of the magnetic force to act on the magnetic beads and enhance the overall radially inward force that drives the beads through the liquid-air interface; (ii) a bead ‘pocket’ above the chambers of the extraction structure that prevents the beads from uncontrolled azimuthal movement; and (iii) special milling conditions during the structuring of the thermoforming master, which prevent the creation of groves and consequently, the entrapment of small bead clusters. The demonstration of the set up was done by comparing a disk- and reference-based extraction of RNA from mosquito homogenates, followed by benchtop real-time RT-PCR. The fact that the (1:10 diluted) eluates lead to similar Ct values, and even better for the disk-based extraction, and with a CV < 10%, is a confirmation of the well-accepted reproducibility of the automated extraction module, especially with complex tissue samples like the mosquito homogenates.

A next step includes the investigation of additional conditions and configurations that will enable the performance of magnetophoresis under rotation with (super-)paramagnetic beads. This could be achieved, for example, by increasing the number of magnets, and/or changing their relative radial positions, with the overall goal to increase the magnetic force on the bead cluster and the space along a full rotation that this force is applied. Furthermore, the extraction structure, interfaced with a downstream nucleic acid amplification module, will be implemented in diagnostically related applications and detailed sensitivity (limit of detection) analysis will be conducted in a sample-to-answer configuration.

## 6. Patents

S.H., N.P., M.R. and K.M. applied for a patent related to magnetophoresis at continuous rotation.

## Data Availability

Data are contained within the article or in the Supplementary Material.

## Supplementary Materials

The following supporting information can be downloaded at: https://www.mdpi.com/article/10.3390/mi13122112/s1, Figure S1: Computational analysis of the magnetic vector potential and the absolute magnetic flux density; Figure S2: Calculation of the bead cluster velocity; File S3: Commercial extraction kits and suppliers; File S4: Liquid properties of various nucleic acid extraction buffers; File S5: Manual mechanical lysis of mosquito pools; File S6: Microfluidic and temperature protocol for running the LabDisk; File S7: LabDisk design and fabrication; Figure S3: Magnetic force vectors at various radial positions; Figure S4: Simulated magnetic force over one full rotation using the initial magnet setup; Figure S5: Design and foto of the magnet installations on the LabDisk processing device; Figure S6: Simulated magnetic force over one full rotation using the improved magnet setup; Figure S7: Magnetic force in radial direction derived from simulated and experimentally measured flux density; Figure S8: Net total force in r-direction (experimental calculations); Figure S9: Inertia acting on a magnetic bead cluster; Figure S10: Improved milling process leading to improved bead transfer. Refs. [53,54] are in Supplementary Materials file.
