# Supplementary material for: Magnetophoresis in Centrifugal Microfluidics at Continuous Rotation for Nucleic Acid Extraction
Source: Micromachines (Basel). 2022 Nov 29;13(12):2112. doi: 10.3390/mi13122112 (PMC9787563; doi:10.3390/mi13122112)
Supplement: Supplementary file 1 [file micromachines-13-02112-s001.zip › micromachines-2000805-supplementary.pdf]

Supporting Information

Magnetophoresis in centrifugal microfluidics at continuous rotation for nucleic acid extraction

File S1: Computational analysis of the magnetic vector potential and the absolute magnetic flux density.

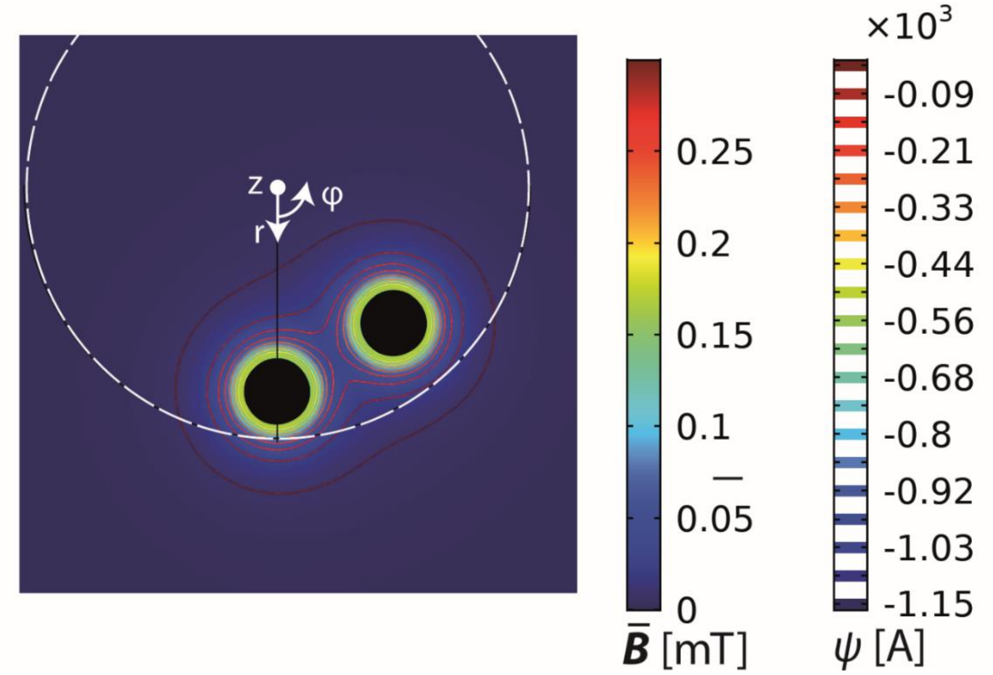

Figure S1. Magnetic vector potential ψ (in A), and the absolute magnetic flux density $\bar{B}$ (in mT) at the r-φ-plane using the initial magnet setup of the manuscript Figure 2. Magnets are shown in black. White dashed line refers to a radial distance of $r = 52$ mm from the center of rotation. Solid lines indicate the magnetic vector potential ψ. Values for $\psi > 0.5$ A are not visible in this image, since this range of magnetic vector potential is found only below the magnets.

File S2: Calculation of the bead cluster velocity

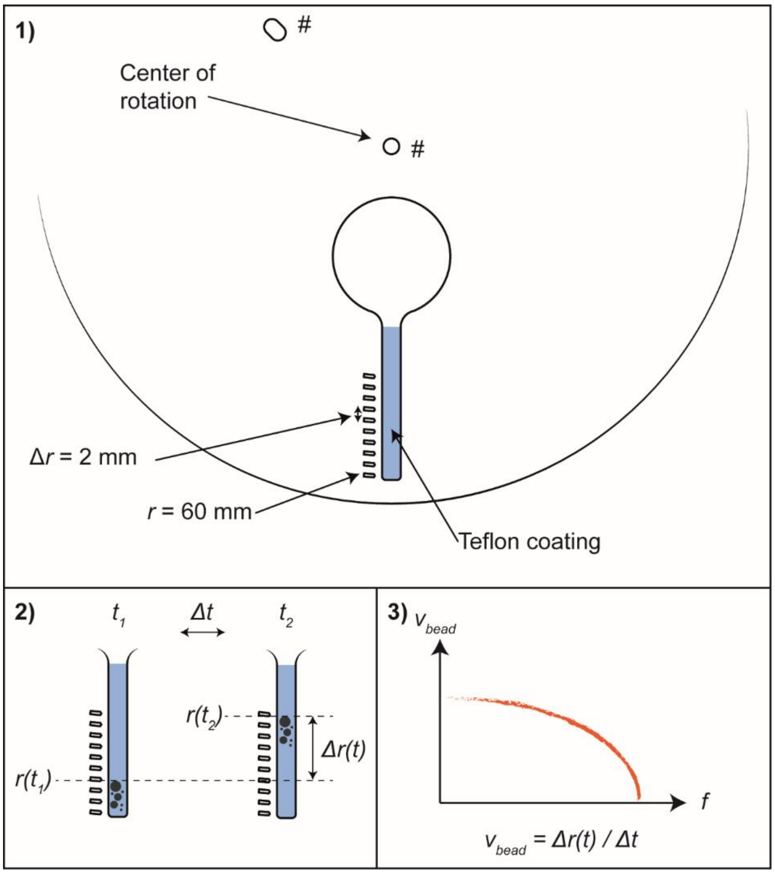

Figure S2. (**1**) Schematic of the setup used for calculating the bead cluster velocity. A rectangular microfluidic chamber on a LabDisk is filled with liquid and magnetic beads suspension. Line markers next to it facilitate the visual assessment of the cluster positon over time. (**2**) The radial distance that the cluster has moved, Δr(t), is determined by means of stroboscopic images, out of which the velocity v_{bead} is calculated. (**3**) The plot of cluster velocity versus different tested rotation frequencies. The symbol # denotes structures for azimuthal orientation during laser cutting.

File S3: Commercial extraction kits and suppliers

List of commercial extraction kits and suppliers.

Kit name	Article number	Manufacturer
Dynabeads® Silane viral NA kit	37011D	Thermo Fisher Scientific Inc., USA
Ethanol assay kit	Z5030029	BioChain Institute Inc., USA
Innuprep MP basic A kit	845-KS-4900100	Analytik Jena GmbH, Germany
MagSi-DNA mf	MD0200010002	MagnaMedics Diagnostics BV (currently magtivio BV), the Netherlands
NucliSENS Lysis buffer	200292	bioMérieux Deutschland GmbH, Germany

File S4: Liquid properties of various nucleic acid extraction buffers

Density and surface tension of various nucleic acid extraction reagents. AJ: Analytik Jena. vNA: viral nucleic acid kit from Thermofisher. BioM: bioMérieux. n = 10 for each datapoint. For reagent details see File S3.

	**Buffer/Mixture**	**Density [kg m^{-3}] (± SD)**	**Surface tension [mN m^{-1}] (± SD)**	**Temperature [°C]**
MagSi DNA mf kit	MMD binding buffer	1056.8 (±15.9)	21.4 (±0.1)	22.9
	MMD lysis buffer	988.4 (±16.5)	31.4 (±0.1)	22.9
	MMD wash buffer 1	898.1 (±27.5)	30.6 (±0.1)	22.5
	MMD wash buffer 2	888.6 (±18.0)	29.8 (±0.3)	22.5
	MMD elution buffer	953.2 (±5.1)	71.5 (±0.2)	22.9
	MMD binding mix*	1022.2 (±22.1)	22.1 (±0.03)	22.5
Innuprep MP basic A	AJ Binding buffer	1060.4 (±35.7)	31.4 (±0.1)	23.2
	AJ lysis buffer	1105.8 (±4.7)	60.6 (±0.8)	23.2
	AJ wash buffer 1	951.4 (±18.5)	32.1 (±0.4)	23.2
	AJ wash buffer 2	783.0 (±5.7)	28.7 (±0.1)	23.2
	AJ binding mix**	1048.8 (±13.4)	32.4 (±0.2)	23.2
Silane viral NA kit	vNA lysis/binding buffer	1068.2 (±12.4)	32.2 (±0.2)	22.9
	vNA wash buffer 1	902.2 (±9.0)	27.9 (±0.5)	22.9
	vNA wash buffer 2	798.5 (±35.4)	30.5 (±0.9)	22.9
	vNA binding mix***	970.4 (±28.2)	29.2 (±0.6)	22.9
	BioM lysis buffer	1100.40 (±16.4)	30.0 (±0.1)	22.5
	AJ/BioM binding mix****	1028.00 (±9.9)	n/a	22.5
	50% (*v/v*) ethanol in water	882.6 (±14.6)	31.3 (±0.7)	22.5

*: MMD (currently magtivio BV) binding mix consists of 19% (*v/v*) MMD lysis buffer, 56% (*v/v*) MMD binding buffer and 25% (*v/v*) ddi water.

**: AJ binding mix consists of 46% (*v/v*) AJ binding buffer, 30% (*v/v*) AJ lysis buffer, and 24% (*v/v*) ddi-water.

***: vNA binding mix consists of 50% (*v/v*) lysis/binding buffer, 25% (*v/v*) isopropylaclohol, and 25% (*v/v*) ddi-water.

****: AJ/BioM binding mix consists of 46% (*v/v*) AJ binding buffer, 30% (*v/v*) bioMérieux lysis buffer, and 24% (*v/v*) ddi-water.

File S5: Manual mechanical lysis of mosquito pools

The insectary at the Swiss Tropical and Public Health Institute, Switzerland, provided specimens of the Kisumu colony that originates from an MRA-762 egg batch provided by BEI Resources. The mosquitoes were preserved in RNA*later* (article No. 76104, QIAGEN GmbH, Germany). After thawing on a lint-free towel, mosquitoes were rinsed with 500 µL of 1× TE buffer in order to remove the RNA*later*. Pools of ten mosquitoes were collected from the tube and were inserted in a new tube with the addition of 50 µL 1× TE buffer, where they were ground using a pestle (article No. 5314493, Omnilab GmbH, Germany) for 30 s. Then, 150 µL lysis buffer (MagSi-DNA mf kit, article No. MD0200010002, MagnaMedics Diagnotics BV – currently magtivio BV, the Netherlands) was added and the mixture was incubated on a thermo mixer (Thermo Mixer C, article No. 5382000015, Eppendorf AG, Germany) for 10 min at 350 rpm and 35 °C. After centrifugation (2 min at 16,000 rcf), 200 µL of the supernatant was pipetted out of the tube and was used for the extractions.

File S6: Microfluidic and temperature protocol for running the LabDisk

Microfluidic protocol for magnetic bead-based nucleic acid extraction utilizing magnetophoresis under continuous rotation. -: Refers to no change in the parameter in this step.

Index	Step	Acceleration [Hz s^{-1}]	Frequency [Hz]	Temperature [°C]	Hold time [s]
1.	Spin sample to lysis chamber	10	-20	-	No
2.	Stickpack pre-heating	-	-	60	180
3.	Open stickpacks	10	-70	-	45
4.	Set reaction temperature	-	-	35	-
5.	Compensate pressure in lysis chamber	10	-30	-	30
6.	Lysis shake mode	10	-25	-	1
7.	Lysis shake mode	10	-20	-	1
8.	Lysis loop (repeat #6, #7)	-	-	-	600
9.	Overpressure valve actuation	10	-12	-	No
10.	Overpressure valve actuation	-	-	50	0
11.	Set reaction temperature	-	-	50	-
12.	Allow beads to break trehalose interface	10	-7	-	2
13.	Allow beads to break trehalose interface	10	-9	-	2
14.	Binding, mixing	10	-8	-	20
15.	Binding, mixing	10	-15	-	1
16.	Binding loop (repeat #14, #15)	-	-	-	600
17.	Magnetophoresis	5	-10	-	20
18.	Magnetophoresis	5	-8	-	5
19.	Magnetophoresis	5	-7	-	5
20.	Magnetophoresis	5	-6	-	5
21.	Magnetophoresis	5	-5	-	90
22.	Magnetophoresis	5	-4	-	90
23.	Magnetophoresis	5	-3	-	60
24.	Spin beads to next chamber	10	-30	-	20
25.	Washing 1, mixing	5	-10	-	20
26.	Washing 1, mixing	10	-15	-	1
27.	Washing 1 loop (repeat #25, #26) 1×	-	-	-	-
28.	Magnetophoresis	5	-10	-	20
29.	Magnetophoresis	5	-8	-	5
30.	Magnetophoresis	5	-7	-	5
31.	Magnetophoresis	5	-6	-	5
32.	Magnetophoresis	5	-5	-	90
33.	Magnetophoresis	5	-4	-	90
34.	Magnetophoresis	5	-3	-	60
35.	Spin beads to next chamber	10	-30	-	20
36.	Washing 2, mixing	5	-10	-	20
37.	Washing 2, mixing	10	-15	-	1
38.	Washing 2 loop (repeat #36, #37) 1×	-	-	-	-

Index	Step	Acceleration [Hz s^{-1}]	Frequency [Hz]	Temperature [°C]	Hold time [s]
39.	Magnetophoresis	5	-10	-	20
40.	Magnetophoresis	5	-8	-	5
41.	Magnetophoresis	5	-7	-	5
42.	Magnetophoresis	5	-6	-	5
43.	Magnetophoresis	5	-5	-	90
44.	Magnetophoresis	5	-4	-	90
45.	Magnetophoresis	5	-3	-	60
46.	Spin beads to next chamber	10	-30	-	20
47.	Elution mixing	5	-13	50	10
48.	Elution mixing	1	-18	50	1
49.	Elution loop (repeat #47, #48)	-	-	-	600
50.	Cool down	-	-	25	-
51.	Stop				

File S7: LabDisk design and fabrication

The design of the microfluidic cartridge was accomplished using SolidWorks2017 (Dassault Systèmes SolidWorks Corp., Waltham/MA, USA). The fabrication was done at the Lab-on-a-Chip Foundry of Hahn-Schickard, using microthermoforming [1] of thin Polycarbonate (PC) foils ("Makrofol® DE 1-1", Covestro AG, Germany) using a WMP30 (WICKERT Maschinenbau GmbH, Germany) thermoforming machine. After the structuring of the polymer foil, the following back-end processing steps were conducted.

Teflon coating was applied on the nucleic acid extraction structures as well as the bead-storage chamber, in order to ensure hydrophobic properties of the surfaces, and according to the procedure described by Strohmeier et al [2] (using a 0.5% *w/w* Teflon solution in Fluorinert FC770), followed by drying at room temperature for 20 min.

Pre-storage of the selected magnetic beads (from the 'Mag-Si DNA mf' kit) was done by means of air drying. The bead suspension was mixed with a solution of 250 mg/mL trehalose (D(+)-trehalose dihydrate) in ddi-water (bead suspension:trehalose solution volume ratio = 2:1). 30 µL of this mixture was manually pipetted into the bead pre-storage chamber of the LabDisk and was left to dry for 1 h at 50 °C in a circumvention oven (SI19, Bibby Scientific Limited, UK).

Production of stickpacks, which are aluminum pouches for pre-storage of liquid reagents [3], was done using a commercial stickpackgaging machine (SBL-50, Merz Verpackungsmaschinen GmbH, Germany), followed by manual positioning them into the dedicated slots in the disk. Quality control was conducted by weighing each stickpack in order to ensure reproducibility of the released volume, and consequently the fluidic and biochemical functionality of the module (acceptable deviation of total weight was ± 5%). The stored volumes were: 150 µL lysis buffer, 440 µL binding buffer, 200 µL washing buffer 1, 200 µL washing buffer 2, and 180 µL elution buffer.

For the RT-PCR, the primers and probes were dry-stored in the reaction chambers from a volume of 10 µL per chamber. Trehalose (D(+)-trehalose dihydrate) was added to a final concentration of 50 mM following recommendations from prior work [4]. For dry-storage of the oligonucleotides, the same conditions were used as for the magnetic beads.

Furthermore, filters (PTFEPET02205, Merck Millipore, Germany) were placed onto the vent holes in the sealing lid to avoid aerosols leaving the cartridge during processing.

Sealing of the cartridge was done using a pressure sensitive adhesive foil (9795R, 3M Corporation, USA) on a HEX01 (JENOPTIK AG, Germany) machine with a pressure of 1.2 bar.

The ready-to-use disks were stored at ambient temperature, in petri dishes containing one desiccant bag to prevent humidity and protection from direct light.

File S8: Magnetic force vectors at various radial positions

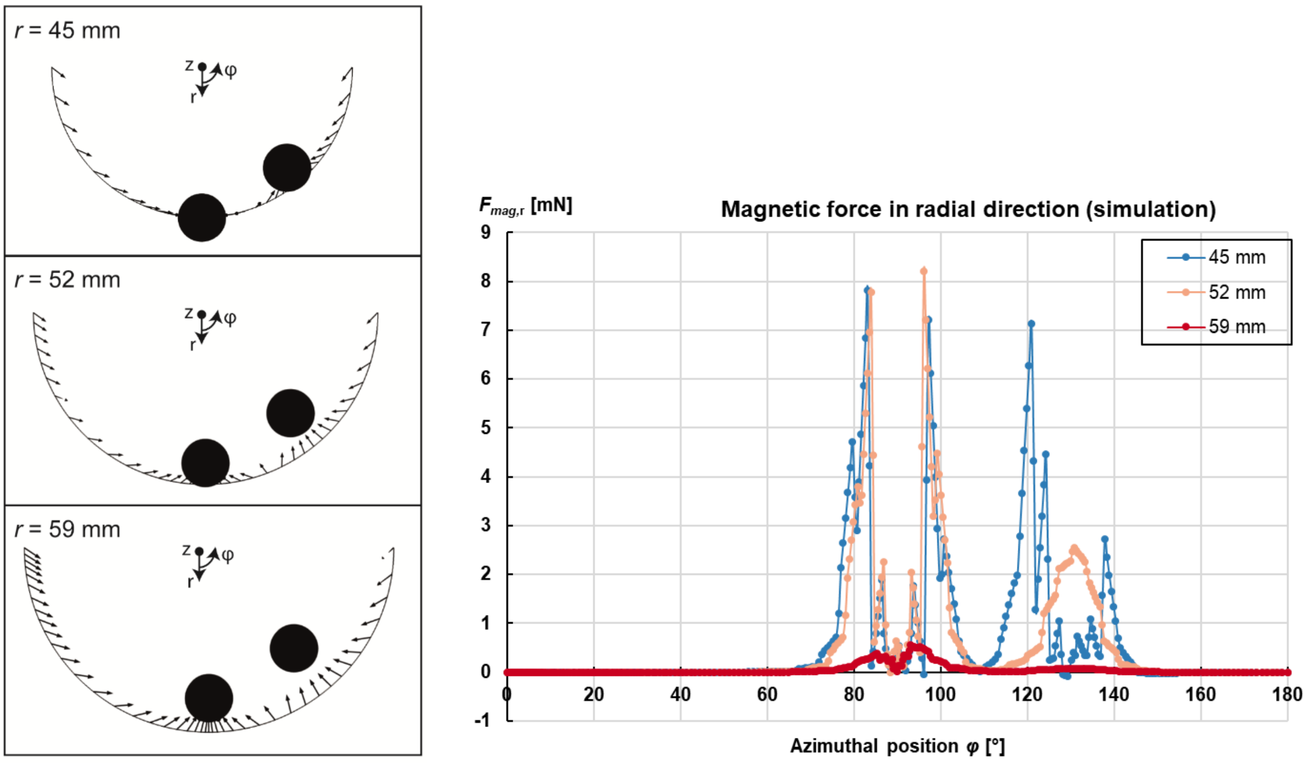

(a) (b)

Figure S3. (**a**): Qualitative representation of magnetic force vectors at various radial positions (*r*), representing typical liquid levels within microfluidic chambers in nucleic acid extraction modules. The vectors' lengths do not scale with their magnitude. (**b**) Simulated magnetic force in radial direction versus the azimuthal position at three radial distances for every value of φ from 0° to 180°. The magnets are arranged according to the manuscript Figure 2. The data was not smoothed.

File S9: Simulated magnetic force over one full rotation using the initial magnet setup

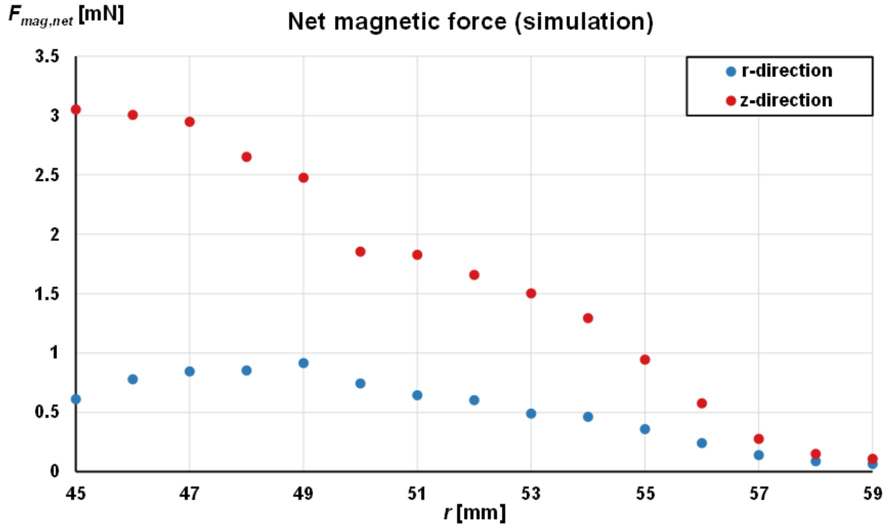

Figure S4. Net (simulated over one full rotation) magnetic force in *r*- and *z*-direction plotted for a range of different radial positions. The data refers to a magnet configuration as shown in manuscript Figure 2 and for a vertical distance of 2.5 mm from the bottom circular surface of the magnets.

File S10: Design and foto of the magnet installations on the LabDisk processing device

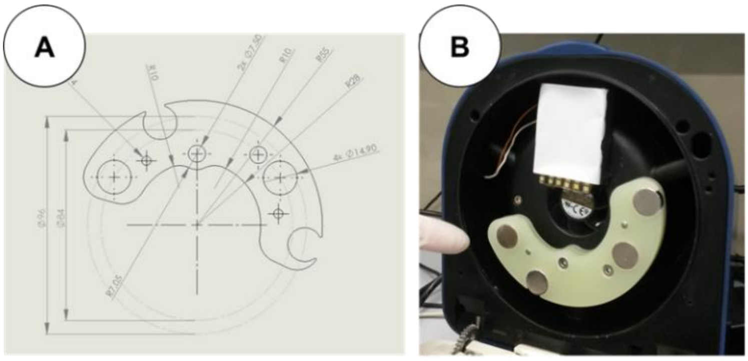

Figure S5. (**A**): Design of the magnet holder. (**B**): Installation of magnets on the lid of the LabDisk processing device (LabDisk Player 1 functional model, QIAGEN Lake Constance – currently Dialunox GmbH, Stockach, Germany).

File S11: Simulated magnetic force over one full rotation using the improved magnet setup

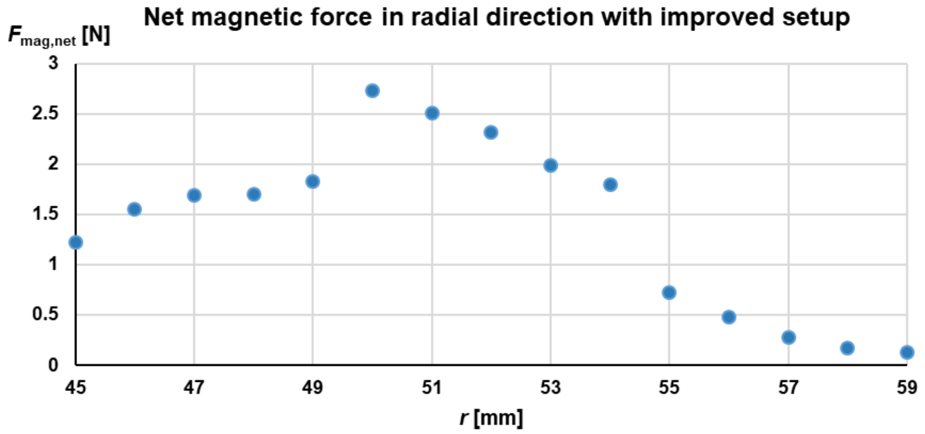

Figure S6. Net (simulated over one full rotation) magnetic force in *r*-direction plotted for a range of different radial positions. The updated configuration of manuscript Figure 3 was used. The contribution of the *z*-component of the magnetic force to the radial bead movement was included, taking into account a tilt angle of $\alpha = 20$ °, $r_{tilt,in} = 50$ mm and $r_{tilt,out} = 54$ mm.

File S12: Magnetic force in radial direction derived from simulated and experimentally measured flux density

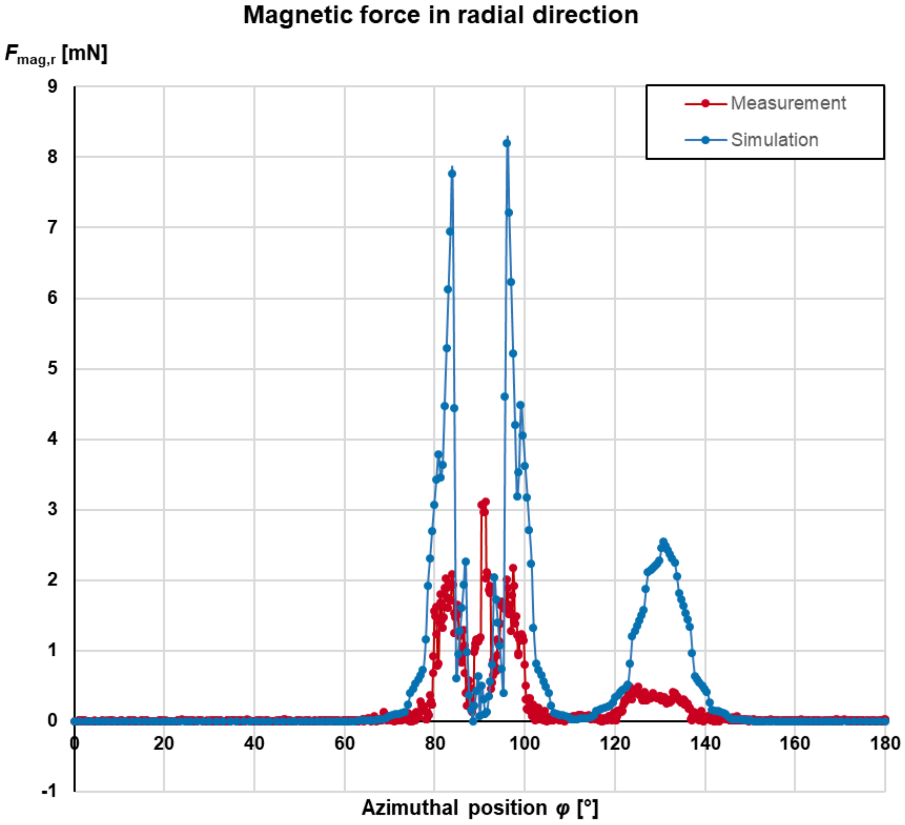

Figure S7. Comparison of magnetic force in radial direction plotted over the azimuthal position, when calculated from simulated flux density versus experimentally measured flux density. The radial distance of 52 mm was used.

File S13: Net total force in r-direction (experimental calculations)

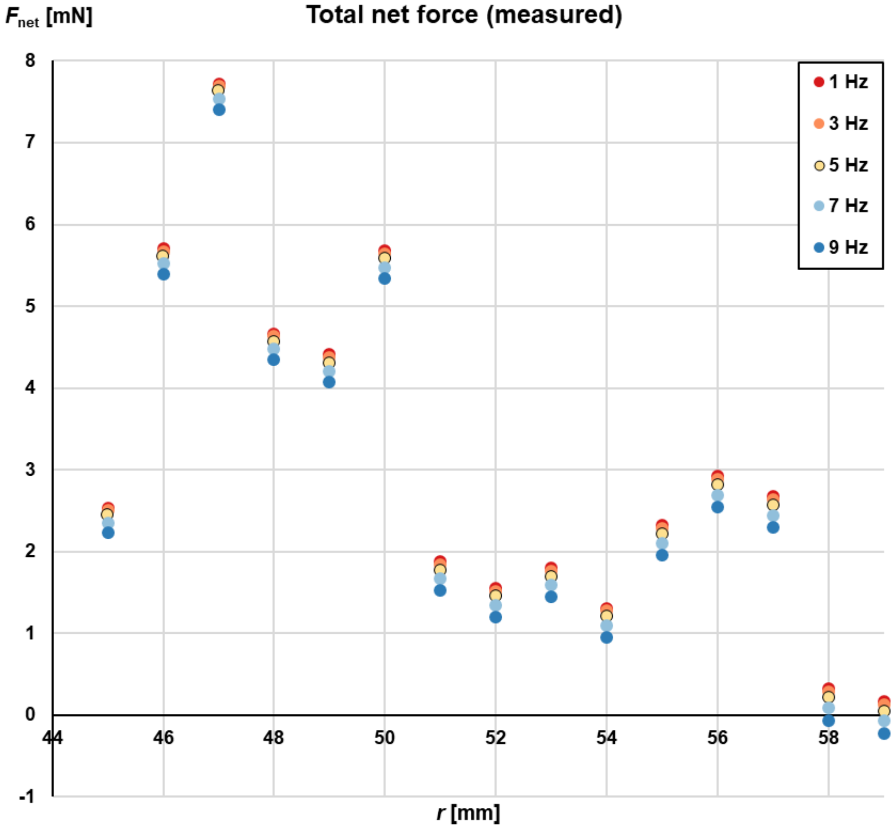

Figure S8. Net (measured over one full rotation based on experimental calculations) total (magnetic, centrifugal and surface) force in *r*-direction plotted for a range of different radial positions and for various rotational frequencies.

File S14: Inertia acting on a magnetic bead cluster

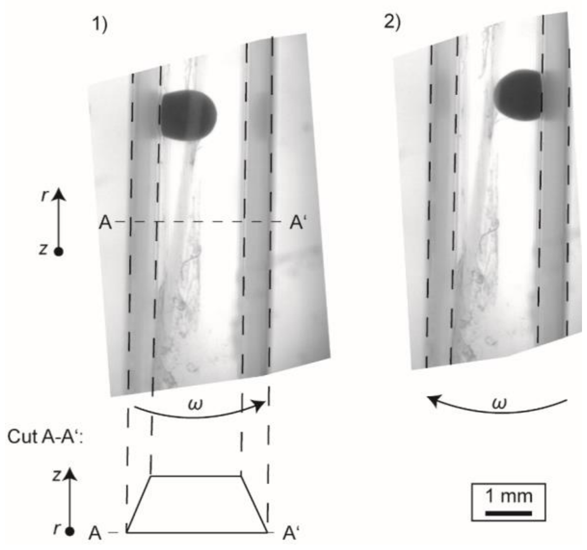

Figure S9. Schematic indicting the act of inertia on a magnetic bead cluster in a radially oriented channel. The counter-clockwise (**1**) or clockwise (**2**) rotation orientation of rotation can define the direction of the bead cluster transfer (here: constrained by a wall in azimuthal direction).

File S15: Improved milling process leading to improved bead transfer

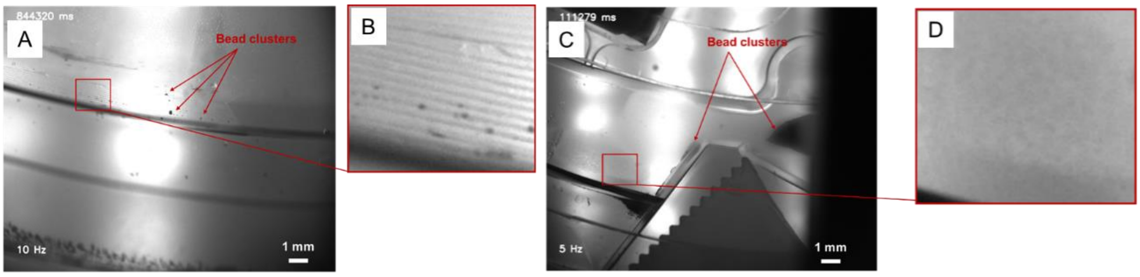

Figure S10. (**A**,**B**): Images of small clusters of beads being trapped in milling groves at the chamber wall. (**C**,**D**): Improved milling process (shorter distance between milling lines), which results in drastic reduction of milling tracks.

References

1. Focke, M.; Kosse, D.; Al-Bamerni, D.; Lutz, S.; Müller, C.; Reinecke, H.; Zengerle, R.; von Stetten, F. Microthermoforming of microfluidic substrates by soft lithography (μTSL): optimization using design of experiments. *J. Micromech. Microeng.* **2011**, *21*, 115002.
2. Strohmeier, O.; Emperle, A.; Roth, G.; Mark, D.; Zengerle, R.; von Stetten, F. Centrifugal gas-phase transition magnetophoresis (GTM) – a generic method for automation of magnetic bead based assays on the centrifugal microfluidic platform and application to DNA purification. *Lab Chip* **2013**, *13*, 146–155.
3. van Oordt, T.; Barb, Y.; Smetana, J.; Zengerle, R.; von Stetten, F. Miniature stick-packaging – an industrial technology for pre-storage and release of reagents in lab-on-a-chip systems. *Lab Chip* **2013**, *13*, 2888.
4. Rombach, M.; Kosse, D.; Faltin, B.; Wadle, S.; Roth, G.; Zengerle, R.; von Stetten, F. Real–time stability testing of air–dried primers and fluorogenic hydrolysis probes stabilized by trehalose and xanthan. *BioTechniques* **2014**, *57*, 151–155.
